# The Microbiology of Olive Mill Wastes

**DOI:** 10.1155/2013/784591

**Published:** 2013-10-03

**Authors:** Spyridon Ntougias, Kostas Bourtzis, George Tsiamis

**Affiliations:** ^1^Department of Environmental Engineering, Democritus University of Thrace, Vas. Sofias 12, 67100 Xanthi, Greece; ^2^Department of Environmental and Natural Resources Management, University of Patras, 2 Seferi Street, 30100 Agrinio, Greece

## Abstract

Olive mill wastes (OMWs) are high-strength organic effluents, which upon disposal can degrade soil and water quality, negatively affecting aquatic and terrestrial ecosystems. The main purpose of this review paper is to provide an up-to-date knowledge concerning the microbial communities identified over the past 20 years in olive mill wastes using both culture-dependent and independent approaches. A database survey of 16S rRNA gene sequences (585 records in total) obtained from olive mill waste environments revealed the dominance of members of *Alphaproteobacteria, Betaproteobacteria, Gammaproteobacteria, Firmicutes,* and *Actinobacteria*. Independent studies confirmed that OMW microbial communities' structure is cultivar dependant. On the other hand, the detection of fecal bacteria and other potential human pathogens in OMWs is of major concern and deserves further examination. Despite the fact that the degradation and detoxification of the olive mill wastes have been mostly investigated through the application of known bacterial and fungal species originated from other environmental sources, the biotechnological potential of indigenous microbiota should be further exploited in respect to olive mill waste bioremediation and inactivation of plant and human pathogens. The implementation of omic and metagenomic approaches will further elucidate disposal issues of olive mill wastes.

## 1. Introduction

Disposal of olive mill wastes is an important environmental problem in olive-oil producing countries since they are generated in huge quantities in a short period of time. Olive oil is mainly produced in the Mediterranean countries, although other producers, such as Argentina, Australia and Chile, are facing the toxic effects of olive mill wastes [1]. The major olive oil producing countries are Spain, Italy, and Greece, with a production of 1150, 560, and 370 thousand tons annually, respectively, followed by Tunisia and Turkey, with an annual production of 160 thousand tons each [2]. Two and three-phase centrifugal decanters are commonly used in the extraction of the olive oil, with two-phase extraction systems gaining ground due to the lower amount of water consumed for the malaxation of the olive paste [3].

Three-phase extraction systems result in the production of olive oil, olive press cake, and a liquid waste, commonly known as olive mill wastewater (OMW), while two-phase decanters produce olive oil and a viscous sludge-like waste, named as alpeorujo in Spanish [4] and abbreviated herein as TPOMW (two-phase olive mill waste). Both types of wastes are characterized by undesirable color and odor, acidic pH, high salt concentration, and total polyphenolics content. In addition, OMWs are characterized by high chemical oxygen demand (COD) values, whereas TPOMW possesses high organic matter and low water activity (*a*_*w*_). The physicochemical properties of OMW and TPOMW are presented in Table 1. Due to the high organic load and the elevated salt and polyphenols content, olive mill wastes are significant sources of environmental pollution. Their effective management is negatively affected by the seasonal operation and the high territorial scattering of the olive mill sites [5].

Olive mill wastes inhibit seed germination and early plant growth [16], alter soil characteristics [17], and create reducing conditions, affecting microbial diversity in soil [18]. In contrast, olive mill waste phenolics may be used in food and chemical industries as natural antioxidants and disinfectants [19, 20]. Olive mill wastes may be used for biopolymer and biogas production as well as being fertilizer/compost and substrate for growing edible fungi [21–24]. Indeed, these wastes consist of a wide range of valuable resources, such as a high proportion of organic and inorganic nutrients, that can be recycled [20, 25].

Several physicochemical and biological treatment approaches have been applied for the degradation and detoxification of olive mill wastes, for example, implementation of advanced oxidation systems, aerobic biotreatment, and anaerobic digestion [21, 26–28], although the review of treatment technologies for both OMW and TPOMW is beyond the scope of this paper.

In this review, the microbiology of olive mill wastes is examined in depth, and special focus is given to: (a) the microbial ecology of olive mill wastes, (b) OMW-induced toxicity, (c) the effects of olive mill wastes on soil microbial communities, (d) the microbial ecology in bioreactors treating olive mill effluents, and (e) the potential biotechnological application of olive mill waste microbiota.

## 2. Microbial Ecology of Olive Mill Wastes

### 2.1. Bacterial Diversity in Olive Mill Wastes

The majority of OMW microbiota are originated from soil and freshwater environments, while fecal bacteria have been also identified [29, 30]. Bacterial community structure is greatly influenced by the specific cultivar from which OMWs are generated [30]. Bacterial communities in OMW generated from various olive-fruit varieties had only 15% of the OTUs identified in common, indicating a cultivar-dependent microbial profile [30]. In all OMW samples examined by Tsiamis et al. [30], the cultured bacterial diversity consisted of members of *Firmicutes*, *Actinobacteria*, *Alphaproteobacteria*, *Betaproteobacteria*, *Gammaproteobacteria* and *Bacteroidetes*, while the implementation of a high density DNA microarray (PhyloChip) revealed a broader diversity, which was dominated by members of all classes of *Proteobacteria*, *Firmicutes*, *Bacteroidetes*, *Chloroflexi*, *Cyanobacteria* and *Actinobacteria*. Members of the phyla *Acidobacteria*, *Planctomycetes*, *Gemmatimonadetes*, *Verrucomicrobia* and the candidate divisions OP3, TM7, AD3, marine group A, and SPAM were minor constituents of the bacterial biota. DNA microarray development enabled researchers to detect microbial sequences from any sample in a parallel and very fast high throughput manner. The studies of Tsiamis et al. [30] and Goberna et al. [31] pioneered the use of such approaches in studying in depth bacterial (PhyloChip) and archaeal (methanogenic) communities (ANAEROCHIP) in OMW and anaerobically digested TPOMW, respectively.

Cultivation and harvesting practice highly affect bacterial community structure in OMW. Bacterial diversity in OMW from “Mastoidis” variety was dominated by fermentative members of *Bacteria*, such as lactic acid (*Lactobacillus* and *Oenococcus* spp.) and acetic acid (*Acetobacter* and *Gluconacetobacter* spp.) bacteria as well as fecal bacteria related to the family *Prevotellaceae* and the *Ruminococcus*-*Eubacterium*-*Clostridium* (REC) cluster [29]. The proliferation of such community structure is attributed to the accumulation of olives in the harvest net which can lead to the establishment of anaerobic/microaerophilic niches, while their processing in olive mills can increase the oxygen level, favoring the growth of acetic acid bacteria [29]. Members of *Betaproteobacteria* (families *Comamonadaceae*, *Gallionellaceae*, *Hydrogenophilaceae*, *Methylophilaceae*, *Oxalobacteraceae* and *Rhodocyclaceae*), *Gammaproteobacteria* (families *Pasteurellaceae* and *Xanthomonadaceae*) and *Firmicutes* (families *Bacillaceae*, *Paenibacillaceae*, *Peptococcaceae* and *Sporolactobacillaceae*) were identified in *O. europaea* var. *koroneiki*-generated OMW [29]. As a result of the early collection and harvesting practice (collection by hand), fermentative bacteria in *O. europaea* var. *koroneiki*-generated OMW were restricted to a few representatives of the families *Peptococcaceae* and *Sporolactobacillaceae*. The identification of fecal bacteria in *Olea europaea* var. *mastoidis*-generated OMW, due to the prolonged harvesting period, is of concern [29].

Vivas et al. [32] found that TPOMW was dominated by members of the phylum *Proteobacteria*, followed by *Actinobacteria* (*Streptomyces*), *Firmicutes* (*Staphylococcus*) and uncultured *Acidobacteria* strains as minor constituents of olive waste microbiota. Members of *Hydrocarboniphaga*, *Pseudoxanthomonas* and *Stenotrophomonas* (*Gammaproteobacteria*) were identified, while *Comamonas* (*Betaproteobacteria*) was the main microbial group detected. Moreover, a *Brevundimonas* sp. was the only representative within *Alphaproteobacteria* [32]. 

In order to face disposal problems related to acidic pH and undesirable odor, addition of Ca(OH)_2_ to TPOMW results in the formation of an alkaline secondary waste (alkaline TPOMW) [4]. This pH change favors alkalitolerant and alkaliphilic bacteria with some degree of halophilicity [4]. Halotolerant alkaliphiles related to the genera *Bacillus*, *Idiomarina*, *Halomonas,* and *Nesterenkonia* as well as alkalitolerant and/or halotolerant bacteria associated phylogenetically with the genera *Corynebacterium*, *Novosphingobium*, *Ochrobactrum*, *Pseudomonas*, *Rhodobacter,* and *Serratia* have been identified in alkaline TPOMW [4]. The majority of those isolates could effectively utilize phenolic compounds as the sole carbon source [4]. Olive pomace microbiota appear to alter their membrane lipids in an atypical manner in order to be adapted to stressful conditions, such as the lowered water activity (*a*_*w*_), the low acidity, and the high polyphenolic content of olive mill wastes [33].

*Staphylococcus* spp. in olive mill waste should be considered as potential infectious agents. Based on microbial counts and API identification, *Enterobacter cloacae* strains were frequently detected in the raw effluent, followed by members of the species *Aeromonas hydrophila*, *Pseudomonas aeruginosa,* and *Serratia odorifera* [34], while *Citrobacter braakii* was predominant during acidogenesis. Representatives of the species *Burkholderia cepacia*, *Enterobacter cloacae,* and *Photobacterium damselae* were also detected. Moreover, high counts of *Acinetobacter*, *Pseudomonas,* and *Enterobacter* spp. have been determined in OMW [34]. Interestingly, *Enterobacter* spp. are potential human pathogens.

**Figure 1:**
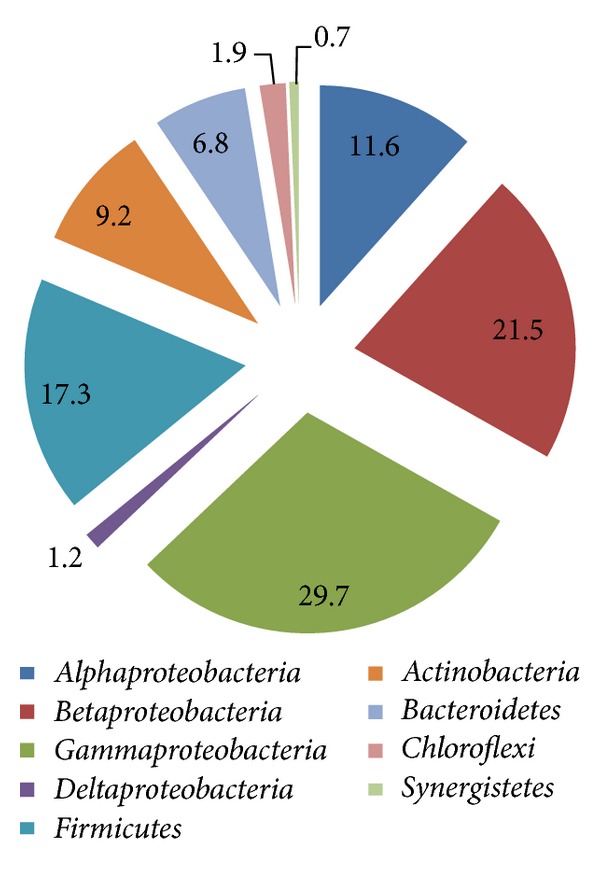
Distribution of bacterial phylotypes identified in olive mill waste environments.

It is concluded that members of *Alphaproteobacteria*, *Betaproteobacteria* and *Gammaproteobacteria* as well as *Firmicutes* and *Actinobacteria* are the main bacterial representatives in olive mill wastes (both OMW and TPOMW). Despite the fact that the distribution analysis of the 16S rRNA gene sequences deposited in international databases may be subjected to bias due to PCR amplification and/or the specific focus of each research work, for example, examination of tannase-expressing communities [35], survey of 16S rRNA gene databases revealed the presence of 585 deposited sequences of bacteria identified in olive mill waste environments. Analysis of these phylotypes confirms the placement of the majority of olive mill waste microbiota in *Alphaproteobacteria*, *Betaproteobacteria*, *Gammaproteobacteria*, *Firmicutes* and *Actinobacteria* (Figure 1).

**Figure 2:**
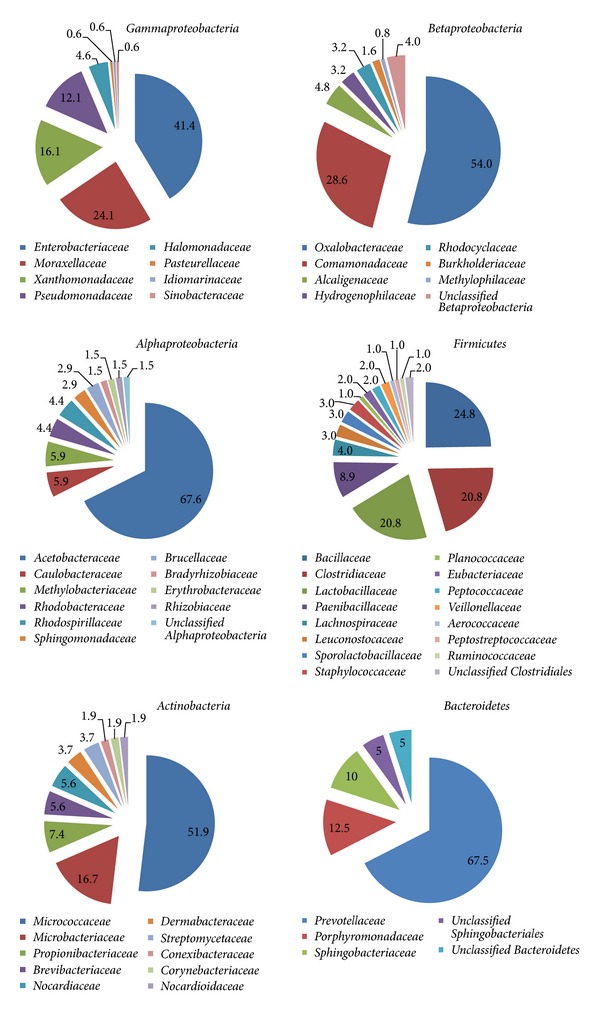
Distribution within *Alphaproteobacteria*, *Betaproteobacteria*, *Gammaproteobacteria*, *Firmicutes*, *Actinobacteria* and *Bacteroidetes* of bacterial phylotypes identified in olive mill waste environments.

The representatives of *Betaproteobacteria* and *Gammaproteobacteria* exceed 50% of the respective 16S rRNA gene sequences deposited in GenBank, while *Firmicutes*, *Alphaproteobacteria*, *Actinobacteria* and *Bacteroidetes* account for approximately 17, 12, 9 and 7% of the bacterial phylotypes identified in olive mill wastes, respectively (Figure 1). Phylotypes' distribution in *Alphaproteobacteria*, *Betaproteobacteria*, *Gammaproteobacteria*, *Firmicutes*, *Actinobacteria*, and *Bacteroidetes* is presented in Figure 2. 

Based on the above analysis (Figure 2), *Enterobacteriaceae*, *Moraxellaceae*, *Xanthomonadaceae* and *Pseudomonadaceae* spp. are the main representatives of *Gammaproteobacteria*. In addition, *Oxalobacteraceae* and *Comamonadaceae* strains are the predominant *Betaproteobacteria* in olive mill wastes, while *Acetobacteraceae* is the dominant taxon within *Alphaproteobacteria*. In olive mill wastes, *Bacillaceae*, *Clostridiaceae*, *Lactobacillaceae* and *Paenibacillaceae *are the most abundant taxa within the phylum *Firmicutes*, while actinobacterial phylotypes were mainly placed in the families *Micrococcaceae*, *Microbacteriaceae* and *Propionibacteriaceae*. *Bacteroidetes* phylotypes identified in olive mill wastes were associated with the families *Prevotellaceae*, *Porphyromonadaceae* and *Sphingobacteriaceae*. Indeed, olive mill waste environments are dominated by bacterial taxa that are specialized in degrading the recalcitrant components of olive mill wastes [29, 30].

Approximately 20% of the bacterial phylotypes identified in olive mill wastes and olive mill waste-related environments are associated with coliforms (e.g., *Citrobacter*, *Escherichia*, *Klebsiella,* and *Serratia* spp.) and other enteric bacteria [36], like *Porphyromonadaceae*, *Prevotellaceae*, *Lachnospiraceae*, *Eubacteriaceae*, *Peptococcaceae*, *Peptostreptococcaceae,* and *Ruminococcaceae* spp. The above findings necessitate the need for safe disposal of OMWs.

### 2.2. Fungal Diversity in Olive Mill Wastes

Yeast population appears to be high in olive mill wastes [17]. Yeasts related to *Geotrichum (G. candidum), Candida (C. membranifaciens, C. michaelii, C. inconspicua, *and* C. tropicalis), Pichia (P. fermentans and P. holstii), Rhodotorula (R. mucilaginosa), *and* Saccharomyces (S. cerevisiae)* have been recently isolated from OMW [37]. In accordance, *Candida boidinii, Pichia holstii *(syn.  *Nakazawaea holstii*)*, P. membranifaciens,* and *Saccharomyces cerevisiae* were the predominant yeasts in OMW from Apulia (Italy), exhibiting high pectolytic and xylanolytic activities. These yeast isolates could effectively reduce total phenolics, resulting in the reduction of several phenolic compounds, in particular *p*-coumaric, vanillic and caffeic acids [38]. *Pichia* (*P. guilliermondii–*syn.  *Meyerozyma guilliermondii*) and *Candida (C. diddensiae and C. ernobii*) spp. were also the main yeast biota in OMW from Moroccan olive mills [39].

*Pichia caribbica (*syn.  *Meyerozyma caribbica), P. holstii (*syn.  *Nakazawaea holstii),* and* Zygosaccharomyces fermentati (*syn.  *Lachancea fermentati*) were the predominant yeast taxa in TPOMW, while *Z. florentinus (*syn.  *Zygotorulaspora florentina), Lachancea thermotolerans (*syn.  *Kluyveromyces thermotolerans), Saccharomyces cerevisiae,* and* S. rosinii (*syn.  *Kazachstania rosinii*) were minor constituents of the yeast community [40]. Some of the yeast isolates from TPOMW exhibited cellulase, **β**-glucanase*, *β**-glucosidase, peroxidase, and polygalacturonase activities which could contribute to the degradation of complex compounds, including olive pomance phenolics [40]. Based on the data provided by Romo-Sánchez et al. [40], yeast diversity in olive pomance appears to be variety dependent.

**Figure 3:**
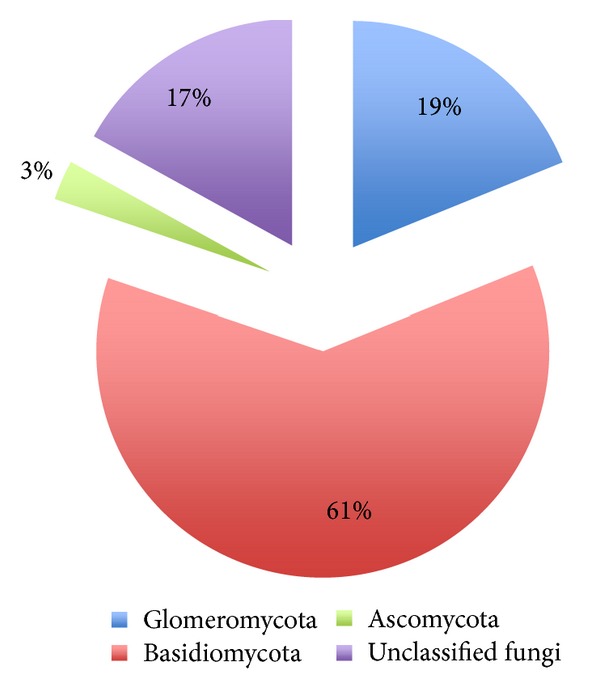
Distribution of fungal phylotypes identified in olive mill waste environments.

Survey of the National Center for Biotechnology Information (NCBI) revealed the presence of 106 deposited sequences of fungi identified in olive mill waste environments. Analysis of these sequences confirms the placement of the majority of fungal phylotypes from olive mill wastes in Glomeromycota, Basidiomycota, Ascomycota, and fungi that have not been assigned (Figure 3). The representatives within the Basidiomycota occupy more than 60% of the fungal phylotypes deposited in GenBank. Members of Glomeromycota and unclassified fungi correspond to 19 and 17% of the respective records, while members of Ascomycota comprise only 3% of the total representatives. However, Ascomycota population is underestimated since Basidiomycota and Glomeromycota specific primers were applied for the majority of the phylotypes identified [41, 42].

Members of the fungal genera *Acremonium*, *Alternaria*, *Aspergillus*, *Chalara*, *Fusarium*, *Lecythophora*, *Paecilomyces*, *Penicillium*, *Phoma*, *Phycomyces*, *Rhinocladiella,* and *Scopulariopsis* have been identified in OMW disposal ponds, possessing the ability to detoxify olive mill effluents [43]. However, the identification of these fungi was based on their morphology and not on molecular techniques. Members of the fungal genera *Cerrena*, *Byssochlamys* (syn.  *Paecilomyces*), *Lasiodiplodia,* and *Bionectria* are indigenous microbiota (identified by molecular techniques) which have the ability to degrade OMW phenolics [44].

Based on the above-mentioned studies, *Pichia*, *Candida,* and *Saccharomyces-*like species are the predominant yeasts in olive mill wastes. Reduction of both phenolics and sugars is the main metabolic function of yeasts in olive mill wastes, while they appear to contribute less in OMW decolorization in comparison to white-rot fungi [39]. Moreover, the acidic pH of olive mill wastes may be advantageous for this microbial group to outcompete bacteria. Filamentous fungi, such as *Aspergillus* and *Penicillium* spp., are common habitants of olive mill wastes [43, 45], while white-rot fungi have been isolated to a lesser extent. It appears that the high salt and sugars concentrations of olive mill wastes as well as the acidic pH favor the growth of osmotolerant yeasts in olive mill wastes [46].

## 3. OMW-Induced Toxicity

Olive mill wastes are toxic to both microorganisms and aquatic organisms. OMWs from traditional mills appear more toxic since their effluents are more concentrated than those from continuous extraction systems [17, 39]. Olive mill effluents negatively affect the aquatic fauna of fluvial ecosystems as a consequence of both high organic load and fecal contamination [47]. Based on Thamnotox kit and zebrafish embryo tests, raw OMW can be characterized as “extremely toxic” and they can retain a significant part of their toxicity even after biotreatment [34]. Moreover, OMW toxicity can reach maximum levels in *Aliivibrio fischeri* bioluminescence assays [48]. The phenolic fraction of olive mill wastes has been reported to exhibit antimicrobial activity against nonindigenous *Bacillus subtilis*, *Escherichia coli*, *Pseudomonas aeruginosa,* and *Staphylococcus aureus* strains, which is even greater than the respective activities induced by the individual phenolic compounds, indicating the synergistic action of olive mill waste phenolics [49]. Experimental data indicate that individual phenolic compounds at low concentrations cannot inhibit the growth of the human pathogens *Escherichia coli*, *Klebsiella pneumoniae*, *Staphylococcus aureus,* and *Streptococcus pyogenes*, while OMWs exhibit strong inhibitory effects against both Gram-positive and Gram-negative bacteria [50]. OMW extracts in combination with gallic acid could effectively inhibit the growth of the above human pathogens at concentrations lower than 100 *μ*g mL^−1^ [50].

In earlier studies, OMW toxicity was attributed to low molecular weight phenolics, in particular monomeric phenolic compounds [51]. However, recent findings have showed that other factors also contribute to OMW acute toxicity, since reduction of monomeric phenolics cannot necessarily lead to mitigation of toxicity [52]. Despite the fact that the mechanism of OMW-induced toxicity remains unclear, several OMW compounds, including phenolics, may cause a narcotic action to seeds and early plants, as the result of a noncovalent membrane interaction [52]. Bioactive intermediate compounds derived from the transformation of phenolics may also be toxic [52]. OMW has been also reported to decrease the phosphorylation efficiency of mitochondria, probably as the result of structural changes induced in the inner mitochondrial membrane by OMW organic compounds (e.g., fatty acids) [53]. Phenolic compounds, such as *p*-coumaric acid and ferulic acid, can also affect the physiology of both prokaryotic and eukaryotic organisms [54]. Moreover, other OMW components, for example, lipids, cannot be excluded. In addition, the low pH and the osmotic stress caused by the presence of high Na^+^ and Cl^−^ concentrations may play a role in OMW acute toxicity [54].

Microbial communities in OMW may be directly involved in acute toxicity mainly against aquatic biota. Venieri et al. [34] reported that specific indigenous microbial taxa, that is, *Aeromonas hydrophila* and *Enterobacter cloacae*, negatively affected the aquatic crustacean *Thamnocephalus platyurus*, linking the microbial activity of certain OMW indigenous microbiota with the toxicity on aquatic organisms. This emphasizes the necessity of assessing microbial communities in OMW, not only for bioremediation purposes but also for safe disposal.

On the other hand, olive mill waste total phenolics content can be used to control and inactivate plant and human pathogens. Yangui et al. [55] revealed the fungicidal action of hydroxytyrosol-rich OMW extract on the soil-borne plant pathogen *Verticillium dahliae*. Moreover, OMW- and TPOMW-phenolic compounds can be used in the inactivation of pathogenic bacteria and their toxins. Administration of the phenolic substrate 4-hydroxytyrosol can inactivate *Staphylococcus aureus* without being cytotoxic to spleen cells, reducing in parallel the biological activity of the staphylococcal enterotoxin A [56]. In addition, the molluscicidal activity of olive mill waste phenolics has been reported [49], while TPOMW extracts have been shown to exhibit suppressive properties against common weeds and nematodes [57].

Several studies have proven the negative effects of these wastes on soil microbial populations, on aquatic ecosystems, and even in air quality [58]. This realization enforces the need for microbial risk assessment during disposal of olive mill wastes. There is, therefore, a necessity for guidelines to manage these wastes through technologies that minimize the environmental impact and lead to a sustainable use of resources.

## 4. Effects of Olive Mill Wastes and Olive Mill Wastes-Derived Composts on Soil Microbiota

### 4.1. Effects of Olive Mill Waste Spreading on Soil Microbiota

Soil microbial activity appears to be enhanced during olive mill waste land application. Controlled OMW spreading can increase the total soil microbial population, which can be accompanied by respective increase in the population abundance of spore-forming bacteria, *Actinobacteria*, and yeasts [59–63]. OMW applications in loamy soils can affect bacterial community structure, due to the high availability of OMW phenolics in this type of soil [64]. By using fatty acids methyl esters (FAME) analysis, Mechri et al. [65, 66] examined the relative abundance of *Actinobacteria* and fungi under successive OMW dose applications. OMW application influences the actinobacterial community structure only in loamy sand soils, indicating soil-dependent effects [18]. Lowered oxidative conditions, availability of phenolics, and N immobilization are the main environmental factors responsible for shifts in microbial communities during OMW spreading [18]. In contrast to the increase of spore-forming bacteria reported in the above studies, lower Gram-positive:Gram-negative FAME quotients were observed during consecutive OMW application [66]. This may indicate a metabolically active Gram-negative bacterial population in comparison to a dormant spore-forming population. The rise in total soil microbial population is also evidenced by the increased soil respiratory activity determined, which is linked to organic substrates degradation and assimilation [60, 67]. Besides, the relative abundance of fungi over bacteria in soils receiving long-term olive mill waste applications can be attributed to the early decomposition of the labile organic matter [68]. In addition, *Actinobacteria* and yeasts as well as several Gram-negative taxa, for example, *Pseudomonas* spp., are considered as effective degraders of recalcitrant compounds [69, 70]. Similarly, prolonged storage of OMW in evaporation ponds increases fungi-to-bacteria ratio [71]. However, apart from the increase in mesophilic population, elevated total coliforms counts are observed during successive OMW applications onto soils [59]. 

Application of TPOMW onto soils results in an increase in fungal diversity and in a consequent decrease in bacterial diversity [72]. Moreover, phospholipid fatty acid (PLFA) analysis in TPOMW-amended soils revealed a gradual decrease of Gram-positive to Gram-negative bacteria, while microbial activity, as determined by dehydrogenase and fluorescein diacetate hydrolase assays, is stimulated in olive mill waste amendments [72].

Extended OMW dose applications can increase the abundance of the soil denitrifying communities, although the nitrifying population is suppressed as a result of the reducing power of OMW phenolics [60]. In particular, ammonia-oxidizing bacteria (AOB) are highly suppressed in the presence of OMW [59]. At the same time, members of cluster 3 of *Nitrosospira* are proliferated [18]. Consequently, inhibition of nitrification process due to OMW applications on soils affects the soil nitrogen cycle [59].

OMW applications highly influence soil basidiomycete communities, although N fertilization alleviates these effects [41]. Changes in basidiomycete community structure are attributed to organic matter addition and N immobilization occurring during OMW spreading [41]. 

OMW application to soils can lead to transient changes in the arbuscular mycorrhizal colonization of *Vicia faba* plants by *Glomus* spp. [42]. However, their population is restored after long-term plant growth. At dose application greater than 30 m^3^ ha^−1^, OMW spreading on olive tree rhizosphere soils has been reported to decrease soil and root 16:1*ω*5 FAME biomarker as well as olive tree photosynthetic rates, indicating that arbuscular mycorrhizal population may be suppressed under high OMW dose applications [65]. This decrease in the relative proportion of 16:1*ω*5 biomarker was attributed to the increased C/N ratio, total P, and phenolics concentrations determined after long-term spread of olive mill effluents [65]. Sampedro et al. [73] stated that the input of arbuscular mycorrhizas to the plants growing in olive mill dry residue amendments depended on the type of the plant and the arbuscular mycorrhizal species.

### 4.2. Microbial Diversity in Olive Mill Wastes-Based Composts and the Effects of Their Amendments on Soil Microbiota

Functional diversity in olive mill wastes has been reported to be relatively low, although increasing during aerobic treatment [32]. In comparison with untreated olive wastes, composting or vermicomposting showed higher dehydrogenase, **β**-glucosidase, and urease activities [32] as a result of the transformation of phenolics. Composted olive mill wastes and byproducts commonly exhibit high extracellular enzyme activities, although lower activities have been determined for enzymes, which function is linked directly to metabolically active microbial cells. Indeed, activities of enzymes that can be extracellularly secreted, such as esterases and ureases, are relatively high [22, 32, 74, 75]. Carbon and nitrogen content in olive mill waste-based composts appears to influence the functional and catabolic diversity of indigenous microbiota [75]. Moreover, Fernández-Gómez et al. [75] showed that TPOMW microbiota possess the ability to oxidize plethora of C substrates.

Composting or vermicomposting can shift bacterial diversity, resulting in the abundance of *Alphaproteobacteria* and *Actinobacteria* in relation to *Betaproteobacteria* [32]. Fernández-Gómez et al. [76] reported that olive-mill waste and biosolids-based vermicomposts were dominated by *Microbacterium*, *Pseudomonas*, *Streptomyces* and *Sphingobacterium* spp. Both *Actinobacteria* and *Sphingobacteria* are involved in the biostabilization of complex compounds. 

A significant part of indigenous microbiota with degrading ability is involved in the initial attack of recalcitrant components of olive mill wastes [35]. Olive mill waste-based composts appear to favor growth of bacterial communities, which are specialized in organic matter decomposition, in particular of phenolics, tannins and lipids. For instance, tannase-expressing bacterial communities, which consisted of members of the phyla *Actinobacteria* (*Kocuria, Microbacterium*, *Micrococcus* and *Rhodococcus* spp.), *Firmicutes* (*Bacillus*, *Lysinibacillus,* and *Staphylococcus* spp.), and *Proteobacteria* (*Acinetobacter*, *Advenella*, *Pseudomonas* and *Pusillimonas* spp.), were identified in TPOMW-based composts [35]. Moreover, various *Bacillus* spp. isolated from OMW exhibit strong lipolytic activities [77].

Olive mill waste-based composts have been proposed to be beneficial in the bioremediation of contaminated soils due to the presence of microbial consortia with degradation ability. Olive mill waste-based vermicompost has been used for the bioremediation of trichloroethylene-contaminated soils through the dominance of bacterial communities related to the phyla *Proteobacteria* and *Acidobacteria*, followed by members of *Bacteroidetes*, *Actinobacteria* and *Gemmatimonadetes* [78]. TPOMW-based vermicompost has been also applied as organic amendment for the bioremediation of PAH (polycyclic aromatic hydrocarbons)-contaminated soils, resulting in changes in bacterial communities which led to enhanced naphthalene dioxygenase activity [79].

## 5. Microbial Community Structure in Bioreactor Systems Treating Olive Mill Wastes

Bacterial diversity has been investigated during acidogenesis of OMW in anaerobic packed-bed biofilm reactors supported with either granular activated carbon or ceramic cubes. Development of acidogenic biofilm in granular activated carbon resulted in a bacterial community structure, which consisted mainly of *Betaproteobacteria* and *Gammaproteobacteria* (*Acinetobacter*, *Comamonas* and *Massilia* spp.), followed by representatives of *Clostridiales*, *Alphaproteobacteria* and *Chloroflexi*. On the other hand, ceramic cubes favored the biofilm formation from *Bacillus*, *Clostridium*, *Paenibacillus* and *Pasteuriaceae* strains [80]. The dominance of *Lactobacillus* and *Acetobacter* spp. in the OMW used [80] indicated that olive mill effluent was naturally fermented during storage. Removal of OMW phenolics through a resin and acidogenesis of permeate in mesophilic anaerobic packed-bed biofilm reactor resulted in the proliferation of bacterial communities consisting almost exclusively of members of *Firmicutes*, that is, *Clostridium*, *Anaerotruncus colihominis*, *Ethanoligenens harbinense,* and *Syntrophobotulus glycolicus* strains [81]. *Actinomyces suimastitidis* and *Staphylococcus felis* were minor constituents of the biofilm formed [81]. Investigation of microbial dynamics in a granular activated carbon packed-bed anaerobic bioreactor fed with OMW resulted in the identification of members of *Gammaproteobacteria*, *Deltaproteobacteria*, and *Bacteroidetes* as well as fermentative bacteria of the genera *Clostridium* (*Firmicutes*) and *Anaerobaculum* (*Synergistetes*) [82]. *Syntrophus* spp., which were in syntrophic relationship with methanogenic *Archaea*, were detected, while sulphate-reducing bacteria were identified as the result of the high sulphate concentration in the OMW digested. Next to *Clostridia*, which are predominant during acidogenesis, *Syntrophus* and *Chloroflexi*-like bacteria appear to be also common inhabitants of phenolic wastewaters [83]. In fact, fermentative *Clostridia* convert phenolic derivatives to benzoate, which is subsequently transformed by *Syntrophus*-like strains to acetate and H_2_/CO_2_ [84]. In syntrophic association, methanogens then form methane from acetate and H_2_/CO_2_. Despite the fact that acetate was the main volatile fatty acid identified, Bertin et al. [82] reported that the hydrogenotrophic *Methanobacterium formicicum* was the predominant archaeon detected. Moreover, Rizzi et al. [85] reported a relative increase/decrease in *Methanomicrobiaceae/Methanobacterium* population by increasing the organic loading rate in an upflow anaerobic filter treating OMW, while no effect on *Methanosaeta*-like population was observed. This indicates that hydrogenotrophic methanogenic population is highly affected by the organic loading rate applied during anaerobic digestion of olive mill wastes, while acetoclastic methanogens remain almost unaffected.

The microbial diversity during treatment of TPOMW-based mixtures in aerated and nonaerated bioreactors has been also investigated [1], with the microbial community structure being studied in a limited number of aerobic treatment systems. Nutrients (N and P) addition could decrease the phenolic content in the aerated bioreactors [1]. The increase in the relative fungal/bacterial ratio was accompanied by high polyphenolic and organic matter reduction [1], indicating that the stimulation of indigenous fungi can effectively detoxify olive mill wastes. This microbial shift permits direct use of the OMW without the need for inclusion of external degraders. Predominant fungi during aerobic treatment of TPOMW-based mixtures were *Penicillium roqueforti*, *Candida norvegica* and *Geotrichum* sp. Nonaerated olive mill waste-derived mixtures were dominated by fungi, which were related to the species *Pichia membranifaciens* and *Cladosporium herbarum* as well as the genera *Ascochyta* and *Geotrichum* [1]. Aerated olive mill waste amendments are commonly dominated by members of *Gammaproteobacteria*, such as *Stenotrophomonas maltophilia* and *Luteibacter* sp., whereas anaerobic *Clostridia* can be also identified [1]. Bacterial diversity in nonaerated olive mill waste-based mixtures consisted mainly of fermentative bacteria belonging to the phylum *Firmicutes*, for example, *Clostridium tyrobutyricum*, *Lactobacillus vaccinostercus*, *Leuconostoc mesenteroides,* and *Sporolactobacillus inulinus*, followed by *Actinobacteria* (*Rhodococcus fascians*, *Clavibacter michiganensis*, *Curtobacterium albidum* and *Frigoribacterium* sp.) and *Gammaproteobacteria* (*Erwinia persicina*, *Stenotrophomonas maltophilia* and *Luteibacter* sp.) [1].

The microbial diversity in an anaerobic continuous stirred tank reactor (CSTR) treating TPOMW was examined under low and high organic loading conditions [86, 87]. At low organic loading rate (OLR), bacterial diversity was dominated by representatives of low G + C Gram-positive bacteria, in particular *Clostridium* spp., although bacterial community structure consisted of members of *Actinobacteria*, *Gammaproteobacteria*, *Bacteroidetes* and *Deferribacteres* under high OLR operational conditions [87]. Moreover, *Chloroflexi* spp. were involved in the anaerobic digestion process of TPOMW [87].

Methanogenic diversity in TPOMW-fed bioreactors under mesophilic conditions appears to be composed by acetoclastic *Methanosaeta* and *Methanosarcina* species. Investigation of methanogenic communities during codigestion of TPOMW and cattle excreta at 37°C and 55°C was carried out by Goberna et al. [31]. Acetoclastic *Methanosarcina* spp. were predominant under mesophilic conditions, although a shift in methanogenic diversity was observed at thermophilic conditions as the result of increased H_2_ pressure, which favored members of the hydrogenotrophic genera *Methanoculleus*, *Methanobacterium,* and *Methanothermobacter*, together with the acetoclastic thermophile *Methanosarcina thermophila*. *Methanoculleus thermophilicus* and *Methanosarcina thermophila* dominated the anaerobic sludge under thermophilic conditions. Moreover, methane production was exclusively carried out by *Methanosaeta* spp. during anaerobic digestion of TPOMW in CSTRs [86, 87].

## 6. Features of Biotechnological Importance in Olive Mill Wastes Microbiota

### 6.1. Biodegradation of Olive Mill Wastes Using Indigenous and Selected Microbial Strains

Basidiomycetous and ascomycetous yeasts, white-rot fungi, and *Aspergillus* and *Penicillium* spp. are commonly used for the *in vitro* dephenolization and/or decolorization of olive mill wastes, whereas bacterial inocula have been also applied. Most of the microorganisms, which are used in the degradation and detoxification of OMW and TPOMW, have been isolated from other environmental sources, although only a few degraders belong to the indigenous microbiota of olive mill wastes.

OMW indigenous bacteria are capable of degrading various single-ring aromatic components of olive mill effluents. Members of *Alphaproteobacteria*, *Betaproteobacteria* and *Gammaproteobacteria*, such as *Comamonas, Ralstonia* and *Sphingomonas* spp., have been reported to degrade OMW phenolics. Ring-cleavage and *o*-demethylation are among the mechanisms involved in the reduction of olive mill waste phenolics [88]. Bacterial strains isolated from other industrial wastewaters or contaminated sites have been also applied for the detoxification of olive mill waste phenolic compounds. Di Gioia et al. [89] exploited the degradation potential of *Ralstonia* sp. LD35 and *Pseudomonas putida* DSM 1868 in the detoxification of OMW phenolics. Furthermore, *Pseudomonas putida* and *Pediococcus pentosaceus* strains which were previously isolated from activated sludge and forest litter could effectively decolorize OMW and remove total phenolics from TPOMW, respectively [90, 91]. *Azotobacter vinelandii* with nitrogen-fixing capacity has been widely used in the bioremediation of OMW [92, 93]. Moreover, indigenous *Klebsiella oxytoca* strains can effectively alleviate the phytotoxic effects of OMW [94].

*Candida cylindracea, C. rugosa, C. tropicalis, Geotrichum candidum, Rhodotorula glutinis, R. mucilaginosa, Trichosporon cutaneum, *and* Yarrowia lipolytica* are yeasts which have been used widely in the bioremediation of olive mill wastes [95–102]. For instance, a *Geotrichum candidum* strain, which was isolated from an aerated pilot-scale bubble column fed with OMW, could dephenolize and decolorize olive mill effluents [103]. Moreover, *Rhodotorula mucilaginosa*, which was isolated from an OMW evaporation pond, could effectively reduce phenolics and COD [97].

The white-rot fungi *Coriolopsis polyzona (*syn.  *Funalia polyzona), Coriolopsis rigida (*syn.  *Coriolopsis floccosa), Ganoderma australe, G. carnosum, Lentinula edodes, Panus tigrinus (*syn.  *Lentinus tigrinus), Phanerochaete chrysosporium (*syn.  *Phanerodontia chrysosporium), Phanerochaete flavidoalba (*syn.  *Phlebiopsis flavidoalba), Phlebia radiata, Pleurotus ostreatus, P. eryngii, P. pulmonarius, P. sajor-caju (*syn.  *Lentinus sajor-caju), Poria subvermispora (*syn.  *Ceriporiopsis subvermispora*), *Pycnoporus cinnabarinus, P. coccineus, Rigidoporus lignosus (*syn.  *Rigidoporus microporus*), and *Trametes versicolor* have been widely used in the detoxification of olive mill wastes [104–113]. In white-rot fungi, laccases and peroxidases as well as radical oxygen species are involved in the significant decrease of olive mill waste phenolics [114, 115]. The ascomycetes *Aspergillus ibericus, A. oryzae* (syn. *Aspergillus flavus* var. *oryzae*), *A. niger, A. terreus, Fusarium graminearum* (syn. *Gibberellazeae*), *F. lateritium* (syn.  *Gibberella baccata), F. oxysporum, Paecilomyces farinosus (*syn.  *Isaria farinosa), Penicillium chrysogenum *and* P. citrinum* as well as the zygomycetes* Mucor racemosus *and* Rhizopus arrhizus* have been also reported to bioremediate olive mill wastes [102, 116–120].

Despite the fact that several selected microbial strains have been used for the degradation of olive mill waste recalcitrant compounds, the examination of those bioremediation agents in sterile OMW- and TPOMW-based media does not necessary mean that they can be effective degraders under ambient conditions. In fact, white-rot fungi are slow growers, and their ability to *in vivo* dominate over other microbial groups is doubted [121]. Indigenous microorganisms, which have been adapted to the adverse conditions of olive mill wastes, are more likely to effectively colonize the effluent. Indeed, *in vivo* experimentation of indigenous microbiota and other selected microorganisms is needed to guarantee their effectiveness in degrading olive mill wastes [93]. Besides, genetic engineering of extracellular oxidases [122], for example, laccases, manganese peroxidases, versatile peroxidases and lignin peroxidases, and/or implementation of enzyme technology approaches, for example, application of enzyme immobilization techniques, can overcome the limitation for effective colonization of olive mill wastes.

### 6.2. Bioconversion Aspects of Olive Mill Waste Microbiota

A biotechnological application of OMW microbiota is the conversion of tyrosol to phenolic compounds of high antioxidant activity. The biotransformation of tyrosol to hydroxytyrosol and 3,4-dihydroxyphenylacetate by a *Halomonas* strain has been stated by Liebgott et al. [123]. Oleuropein present in olive mill wastes can be also transformed into hydroxytyrosol [124, 125]. Furthermore, phenol-tolerant *Enterobacteriaceae* strains isolated from OMW possess the ability to bioconvert xylose to ethanol [126]. In addition, *Clostridium bifermentans* TYR6 is an anaerobic bacterium which was isolated from OMW and can covert cinnamic acid to 3-phenylpropionic acid [127]. Enhanced **β**-glucan synthase activities have been exhibited by mushroom species growing in OMW [128]. Moreover, *Paracoccus thiocyanatus* and *Halothiobacillus neapolitanus* strains are sulfur-oxidizing bacteria which have been isolated from alkaline TPOMW-based compost and can be used in compost acidification [129]. 

### 6.3. Plant Disease-Suppressive Properties of Olive Mill Wastes

OMWs exert antifungal activity against plant pathogens. OMW can suppress the soil-borne pathogens *Rhizoctonia solani* and *Fusarium solani* at low dose applications [130] as the result of the antimicrobial action of OMW phenolics. In addition, control of Botrytis fruit rot on strawberries and peppers by raw OMW has been also reported [131]. Moreover, OMW application on fruits and vegetables can inhibit the sporulation of *Penicillium* and *Botrytis* spp. and suppress the phytopathogenic effects of *Fusarium oxysporum* f.sp. *lycopersici* on tomato plants [132]. OMW can also inhibit the growth of the seed-borne pathogens of tomato plants, *Clavibacter michiganensis* subsp. *michiganensis,* and *Pseudomonas syringae* pv. *tomato* [133]. Extract from TPOMW-derived composts can suppress the plant pathogen *Pythium aphanidermatum* [134].

Some bacteria isolated from OMW have been reported to exhibit antagonistic effects against soil-borne pathogens. Bacterial strains related to the genus *Bacillus* and the species *Burkholderia caryophylli* and *Pseudomonas fluorescens* induced *in planta* disease suppressiveness against Fusarium and/or Rhizoctonia damping-off of tomato [130]. Moreover, bacterial strains, which were isolated from OMW and related to the species *Bacillus subtilis*, *B. pumilis*, *Pseudomonas putida,* and *Stenotrophomonas maltophilia*, exerted *in planta* antimicrobial activity against *Agrobacterium tumefaciens* [135]. *Serratia marcescens* strain BR2.1 is a biocontrol agent with *in planta* antimicrobial activity against *Fusarium oxysporum* f.sp. *radicis-lycopersici* which was isolated from the rhizosphere of tomato plants growing in OMW-derived compost amendments [136]. TPOMW and TPOMW-derived compost extracts appear to exert a general suppression against soil-borne oomycete *Phytophthora capsici* [137]. However, TPOMW-induced suppression against *Pythium ultimum* and *Botrytis cinerea* was weak and depended on the specific olive mill waste tested, while only mature compost elicited protection against the above-mentioned plant pathogens [137]. TPOMW and TPOMW-based compost extracts could not suppress *Rhizoctonia solani* [137]. In contrast, Bonanomi et al. [138] reported that the radial growth and the hyphal density of the plant pathogens *Fusarium oxysporum* f.sp. *lycopersici*, *Sclerotinia minor,* and *Botrytis cinerea* were increased in olive mill dry residue-amended soils.

Actinobacterial strains, which were related to the genera *Streptomyces* and *Lechevalieria* and isolated from TPOMW-derived compost, exerted suppressive action against fungal and oomycete pathogens, that is, *Fusarium oxysporum* f. sp. *melonis*, *Phytophthora cinnamomi*, *Pythium debaryanum*, *Sclerotinia sclerotiorum,* and *Thanatephorus cucumeris*, and the bacterial strain *Agrobacterium tumefaciens* CECT 4119 [139].

Competition for nutrients and ecological niches, antibiosis (e.g., secretion of volatile metabolites or other antimicrobial agents), spore germination, germ tube elongation inhibition, and lysis via hydrolytic enzymes can be involved in the suppression of soil-borne pathogens by olive mill waste-derived compost amendments [130, 140, 141]. Indeed, a microbe-induced suppresion associated with the dominance of copiotrophs and/or the proliferation of certain microbial groups appears to contribute significantly to OMW and TPOMW suppressive effects [28, 67].

## 7. Conclusions

**Figure 4:**
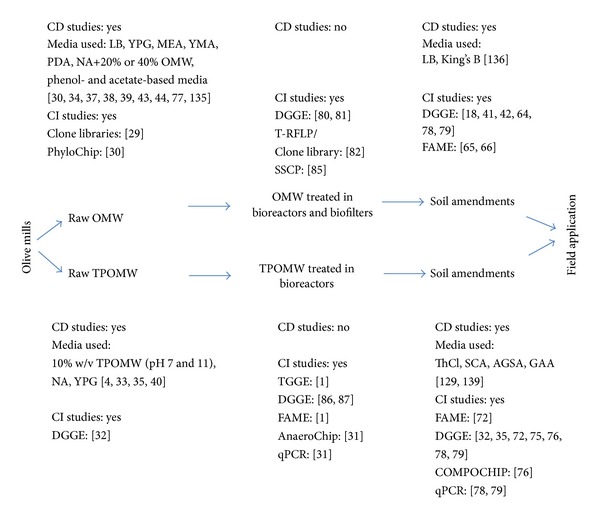
Schematic representation of the olive mill waste route in relation to the studies completed and the level of analysis provided. LB: Luria Bertani medium; YPG: yeast extract-peptone-glucose medium; MEA: malt extract agar; YMA: yeast-malt agar; PDA: potato dextrose agar; NA: nutrient agar; ThCl: medium 152 for *Thiobacillus* (ATCC); SCA: starch casein agar; AGSA: arginine glycerol salts agar; GAA: glycerol asparagine agar. Research studies, considering less than 5 isolates, are not included; CD: culture-dependent, CI: culture-independent.

Monitoring of microbial communities is one of the most fundamental tasks to understand any bioremediation process. Although the importance of monitoring microbial diversity has been extensively stated, only a few studies focusing on the identification of microbial communities in olive mill wastes have been performed. Indeed, such research studies enable an in-depth analysis of olive mill waste biotrasformations. For instance, the implementation of 16S rRNA gene clone libraries and high density DNA microarray (PhyloChip) not only enhanced our knowledge on OMW indigenous microbiota but also established the presence of a cultivar-specific effect [29, 30]. As shown in Figure 4, a limited number of molecular studies, which permit the identification of both cultured and uncultured microbial communities, have been carried out, and no studies have been performed by applying high-throughput techniques, such as pyrotag sequencing and metagenomic approaches. Indeed, implementation of omic approaches, such as high density 16S rRNA microarray (PhyloChip) and 16S rRNA pyrotags, in the fruits of the most important olive-tree varieties and their olive mill-generated wastes, in combination with the examination of the complex nature of olive mill waste phenolics and other physicochemical and environmental features, will elucidate through canonical correspondence analysis the parameters affecting microbial ecology in olive mill effluents. 

The biotechnological potential of olive mill wastes has not been fully exploited since novel bacterial taxa are still being identified in olive mill wastes [142, 143], indicating that there are still unexhausted sources for biotechnology. Genome sequence analyses of indigenous microbiota will reveal the biodegradation pathways of recalcitrant compounds present in olive mill wastes and their adaptive mechanisms to olive mill waste phenolics. Interestingly, the genome sequence analyses of *Olivibacter sitiensis* and *Clostridium methoxybenzovorans* are ongoing, and novel fundamental results are expected. Isolation of novel biocontrol agents with suppressive properties against the major plant pathogens is another experimental task. Indeed, experimentation on new approaches for the cultivation of novel microorganisms and new biocontrol agents deserve further examination.

We propose that the focus of the research should shift from the simple characterization of the microbial communities that are present in OMWs to their functional role, starting with the genome sequencing of important isolates that have been identified in OMWs and/or they have been characterized as degraders. This effort should not only be restricted to strains that have been isolated, but also to uncultured ones that can be characterized through the use of single cell genomic (SCG) approach. Genomes obtained through a SCG approach have been reported for marine bacterial strains and for insect endosymbionts [144–147], and this presents a unique opportunity to identify the metabolic features, the timeline of species evolution, and the inter-organismal interactions of the uncultured microbial groups that dominate the olive mill wastes. Once this is achieved, in-depth metagenomic approaches could be used to characterize the gene repertoire present in OMW, while the genomes sequenced would provide the backbone for mapping the majority of these genes. Metatranscriptomic approaches will further reveal the active microbial communities and the genes that characterize these environments. 

## Figures and Tables

**Table 1:** Basic OMW and TPOMW physicochemical characteristics.

Characteristic (values range)	OMW^1^	Characteristic (values range)	TPOMW^2^
pH	4.01–5.93	pH	4.86–6.45
BOD (g L^−1^)	8.0–38.7	OM (%)	49.5–98.5
COD (g L^−1^)	28.6–186	Total phenolics (%)	0.5–2.4
TOC (g L^−1^)	1.89–38.0	Total N (g kg^−1^)	7.0–18.5
TS (g L^−1^)	3.13–78.2	Total P (g kg^−1^)	0.5–2.2
Total phenolics (g L^−1^)	0.03–18.9	Total K (g kg^−1^)	6.3–29.7
Total N (g L^−1^)	0.02–2.10		
Total P (g L^−1^)	0.01–1.00		
Total K (g L^−1^)	0.17–7.81		

^1^Based on data reported in Aktas et al. [6], Ammary [7], Zenjari et al. [8], Amaral et al. [9], Eroğlu et al. [10], Aviani et al. [11], and Ntougias et al. [12].

^2^Based on data reported in Alburquerque et al. [13], Vlyssides et al. [14], and Baeta-Hall et al. [15].
